# Current News

**Published:** 1905-01

**Authors:** 


### Current News.

### PENNSYLVANIA ASSOCIATION OF DENTAL SURGEONS.

The fifty-eighth annual meeting of the Pennsylvania Association of Dental Surgeons was held at the Continental Hotel, Philadelphia, October 11, 1904. The following officers were elected for the ensuing year: President, Dr. M. I. Schamberg; Vice-President, Dr. Albert N. Gaylord; Secretary, Dr. J. Clarence Salvas; Treasurer and Librarian, Dr. Wm. H. Trueman.

J. Clarence Salvas,
*Secretary.*

